# Gastrointestinal Dystonia in Children and Young People with Severe Neurological Impairment & Palliative Care Needs: A Systematic Review

**DOI:** 10.3390/children12101359

**Published:** 2025-10-09

**Authors:** Timothy Warlow, Jill Yates, Naomi Taylor, Gemma Villanueva, Bindu Koodiyedath, Fiona McElligott, Susie Holt, Anna-Karenia Anderson

**Affiliations:** 1 Department of Specialist Paediatric Palliative Care, University Hospitals Southampton & Naomi House and Jacksplace Hospices, CG63 Southampton General Hospital, Tremona Road, Southampton SO166YD, UK; 2 NHS Lothian, Edinburgh EH4 2XU, UK; jill.yates@nhs.scot; 3 Martin House Children’s Hospice, Wetherby LS23 6TX, UK; naomi.taylor8@nhs.net; 4 Cochrane Response, London W1G 0AN, UK; gvillanueva@cochrane.org; 5 Northampton General Hospital, Northampton NN1 5BD, UK; b.koodiyedath@nhs.net; 6 Children’s Health Ireland, D01 YC67 Dublin, Ireland; fiona.mcelligott@childrenshealthireland.ie; 7 Alder Hey Children’s NHS Foundation Trust, Liverpool L14 5AB, UK; susieholt@nhs.net; 8 Royal Marsden Hospital NHS Trust, London SW3 6JJ, UK; annakarenia.anderson@nhs.net; 9 Shooting Star Chase Hospice, Addlestone KT13 9UQ, Surrey, UK

**Keywords:** gastrointestinal dystonia, gastrointestinal failure, gastrointestinal symptoms, feed intolerance, palliative care, palliative medicine, end of life care, severe neurological impairment

## Abstract

**Highlights:**

**What are the main findings?**

- This study is the first systematic review of the evidence for the management of gastrointestinal dystonia in patients with palliative care needs.
- High certainty evidence is currently lacking, but a significant body of indirect ev-idence exists upon which initial suggestions for practice and further study can be based.

**What is the implication of the main finding?**

- Gastrointestinal dystonia is a heterogeneous condition for which a well co-ordinated, holistic, multidisciplinary approach to management is required.
- Clear identification of goals of care, use of advance care planning, and a rational, systematic approach to symptom management are the mainstay of the treatment approach.

**Abstract:**

Background: Increasing numbers of young people with severe neurological impairment are suffering from gastrointestinal symptoms, which may result in nutritional failure and ultimately death. Gastrointestinal dystonia is a recently described clinical diagnosis amongst patients with severe neurological impairment, and no systematic review of existing evidence currently exists. Aim: To conduct a systematic review of existing evidence for the management of gastrointestinal dystonia in children and young people with severe neurological impairment and palliative care needs. Method: A systematic review assessing pharmacological and non-pharmacological treatments was undertaken using standard Cochrane methodology. We searched Cochrane CENTRAL, MEDLINE, EMBASE, and PsycInfo. All databases were searched from inception, and no language restrictions were used. Results: 1580 references were identified. After abstract screening, 56 references were reviewed at full text, and a case report and case series were identified for inclusion. Low-quality, indirect evidence exists for the management of gastrointestinal dystonia, including symptom management, hydration and nutrition decisions, and end-of-life care. Conclusions: There is a paucity of existing evidence directly relating to gastrointestinal dystonia, but low-quality indirect evidence from studies of children with severe neurological impairment and gastrointestinal symptoms exist, which may begin to inform clinical practice.

## 1. Introduction

In recent years clinicians from the fields of paediatric and adult neurology, gastroenterology, palliative medicine, and community paediatrics have anecdotally reported a group of children and young people with severe neurological impairment developing increasing gastrointestinal problems. The increase noted in this population may be due to the survival of children born at the margins of viability and improved neonatal, neurological and respiratory care of those with neurological impairment who would have previously died earlier in childhood [1]. With these children frequently surviving into the second and third decades, there is a tendency for the gastrointestinal tract to be the source of distress and impairment of quality of life, with nutritional failure an increasingly common mode of death in this group. Symptoms are varied and come in complex clusters.

**Figure 1:**
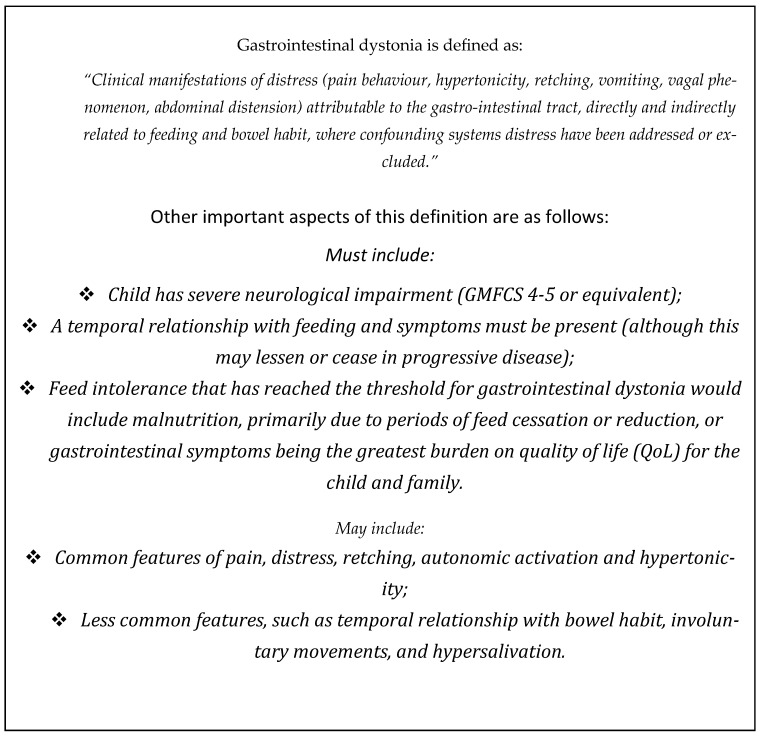
Definition and key features of gastrointestinal dystonia from the appropriateness panel on the ‘Definition, Investigation and Management of Gastrointestinal Dystonia in Children and Young People with Neurodisability’ (2019–2022) [2].

A core group of paediatric palliative care physicians from the Association of Paediatric Palliative Medicine (APPM), worked alongside colleagues in paediatric neurology, gastroenterology, dietetics, surgery, and community paediatrics to develop a previously published consensus definition for this phenomenon outlined in Figure 1 [2].

Children and young people with gastrointestinal dystonia (GID) may have previously been referred to as having ‘gastrointestinal failure’, but their presentation and mode of deterioration do not adhere to the definition of gastrointestinal failure used in the field of gastroenterology [3]. Katz et al. suggest the term ‘intractable feed intolerance’ for this group of patients [4]. While this does capture the predominant presentation in this group, some children present with mainly lower gastrointestinal symptoms, similar to mechanical bowel obstruction; others predominantly retching and vomiting; and others pain and irritability. Therefore, the presentation is more heterogeneous and complex than this definition alludes to. It also fails to capture the feature that sets these children apart from those with short gut syndrome or neuropathic gut disorders—the fact that severe neurological impairment is an intrinsic part of the diagnosis. While we acknowledge the term gastrointestinal dystonia is not a perfect description in terms of the underlying pathological processes, it does ensure this group of children are given a diagnosis in their own right and avoids confusion with other forms of persistent feed intolerance within gastroenterology. Due to their symptoms and distress, patients with gastrointestinal dystonia are unable to tolerate enteral feeding, leading to nutritional insufficiency and, in some cases, failure of enteral nutrition and death [5].

The Association of Paediatric Palliative Medicine (APPM) identified the need for a robust systematic review of existing evidence to inform the non-pharmacological and pharmacological management of gastrointestinal dystonia in children and young people with severe neurological impairment and palliative care needs. Given the novel nature of the diagnostic label, a broad study question and review design would be required, incorporating indirect evidence from gastroenterology and neurology as well as pain and palliative care fields.

### 1.1. Review Question

Which non-pharmacological and pharmacological interventions are effective for the practical management of gastrointestinal dystonia symptoms in children with severe neurological impairment and palliative care needs?

### 1.2. Population

Children with severe neurological impairment and gastrointestinal dystonia, benefiting from a palliative care approach. This might be defined by complexity, perceived benefit of intervention, quality of life, route of drug administration, place of care, or phase of illness.

### 1.3. Interventions

#### 1.3.1. Pharmacological

- Omeprazole, lansoprazole, ranitidine, famotidine, domperidone, and Gaviscon;
- Metoclopramide, erythromycin, levomepromazine, cyclizine, ondansetron, granesitron, stemetil, nabilone, other cannabinoids, aprepitant, and baclofen;
- Gabapentin, pregabalin, amitriptyline, clonidine, SSRI- Fluoxetine, Duloxetine, diazepam, midazolam, lorazepam, clonazepam, clobazam, and chloral hydrate;
- Opioids (morphine, fentanyl, oxycodone, dihydrocodeine, and buprenorphine, methadone) and ketamine;
- Lactulose, movicol, enemas, docusate, sodium picosulfate, and senna;
- Alimemazine, octreotide, Neostigmine, pyridostigmine, cyproheptadine, H. Pylori treatment, and probiotics;
- Over-the-counter remedies: [peppermint tea/oil];
- PN/TPN, home TPN/PN, and fluids IV/SC.

#### 1.3.2. Non-Pharmacological

- Pyridostigmine treatment, Farrell bag, flatus tube, Replogle tube, nasogastric feeding, jejunal feeding, and gastrostomy venting;
- Hydrolysed formulas, alterations of feeding regimen, blended diet, exclusion diets, feed thickeners, and carbogel;
- Psychological intervention, distraction therapy, music therapy, art therapy, play therapy, complementary therapies, acupuncture, hydrotherapy, reflexology, and abdominal massage;
- Place of care, access to tissue viability, bed and seating cushions, mattresses including airflow, oral care and hygiene, over feeding, formula osmolarity, and feeding rate reduction.

#### 1.3.3. Comparison

- Placebo, no treatment/usual care, cross comparison between any of the above (within group and between group), combinations of the above—reducing triggers and pharmacological management, routes of administration (same drug or same drug class).

#### 1.3.4. Outcomes

- Reduced frequency or intensity of gut-related symptoms (pain, nausea, vomiting, retching, bloating, gastric losses, constipation, or diarrhoea);
- Reduced distress as experienced by child and family;
- Supporting individualised family choice around the most appropriate use of hydration and nutrition;
- Establishing new goals of care and accepting changes in care goals;
- Potential improvement in gut motility and/or improved feed tolerance;
- Care in place of choice;
- Improved patient and family experience/carer satisfaction.

The PICO question was reviewed by the external stakeholders to ensure a comprehensive intervention list was obtained and outcomes agreed upon.

## 2. Materials and Methods

A study development group of 42 stakeholders was established consisting of doctors, nurses, and pharmacists working with children and young people with life-limiting conditions across hospital, hospice and community settings from around the United Kingdom. The group also included two parents of children with GID and one patient with another life-limiting condition but extensive experience with hospital, hospice, and community services. The purpose of this group was to ensure a detailed understanding of the issues facing children with GID, provide the ability to assess the appropriateness of both direct and indirect evidence identified for the management of gastrointestinal dystonia, and to ensure that any suggestions for practice emerging from the evidence were developed with the child and family at the centre of that approach. External review of the study proposal was provided by representatives from the British Society of Paediatric Gastroenterology, Hepatology, and Nutrition (BSPGHAN), British Paediatric Neurology Association (BPNA), and British Association of Paediatric Surgeons (BAPS), as well as a senior dietitian. A systematic reviewer and guideline methodologist from Cochrane response provided orientation and an overview of evidence-informed guideline development processes.

MEDLINE (Ovid), Embase (Ovid), and Cochrane CENTRAL (Wiley) were searched in April 2021, and the searches were updated in November 2022 (see search strategy in Supplement S1). All databases were searched from inception, and no language restrictions were implemented. Given the novel definition ‘gastrointestinal dystonia’, the term ‘gastrointestinal failure’ was also used as a primary search term. Screening, data extraction, and risk of bias assessments were performed in duplicate by two independent reviewers. Studies were included that reported on children with severe neurological impairment (global motor function classification system 4–5 or equivalent) and with life limiting conditions as determined by the study authors. Studies were excluded where the majority of subjects were 19 years or older or subjects were diagnosed with mechanical bowel obstruction, e.g., tumour compression. Studies reporting on surgical interventions for management of gastrointestinal dystonia were also excluded.

Following the initial scoping review, the team was aware evidence was likely to be sparse and of low quality; therefore, alongside the formal process, hand searching to identify indirect evidence that related to the management of gastrointestinal dystonia was undertaken. Relevant indirect evidence from the fields of gastroenterology, neurology, and community paediatrics was collated by the study team through reference and citation searches of the original systematic review. This indirect evidence may not have made direct reference to the term gastrointestinal dystonia due to the recent defining of this term. However, indirect evidence collated was subject to the same inclusion and exclusion criteria as the main search and assessed and extracted by two independent reviewers in the same way. Methods were compliant with the Preferred Reporting Items for Systematic Reviews and Meta-Analyses extension for Scoping Reviews (PRISMA-ScR) checklist (Supplement S2), and this process of indirect evidence inclusion is in line with guidance developed by the Royal College of Paediatrics and Child Health [5]. The study was registered on the international prospective register of systematic reviews PROSPERO (registration CRD420251154818).

Due to the lack of quantitative data identified, the use of statistical analysis was not appropriate. The indirect evidence was reviewed extensively by the broad and experienced study development group in order to discern the quality of the evidence and applicability of the evidence to this patient group, in line with recommendations from the Grading of Recommendations Assessment, Development and Evaluation (GRADE) group and Royal College of Paediatrics and Child Health of the United Kingdom [6].

### Developing Suggestions for Practice and Further Study

Results of the systematic review were collated in summary of evidence tables and presented to the study development group. The available evidence was discussed for each intervention in line with the ‘evidence to decision process’ set out by the GRADE group. This process provides a transparent and pragmatic method for developing recommendations by evaluating factors like risk of bias, imprecision, and consistency to determine the certainty of evidence. This evidence-based assessment then informs the strength of the resulting recommendations or suggestions for practice and further study, which can be a simple yes/no or a conditional recommendation, depending on the balance of benefits, harms, costs, and values [7].

## 3. Results

**Figure 2:**
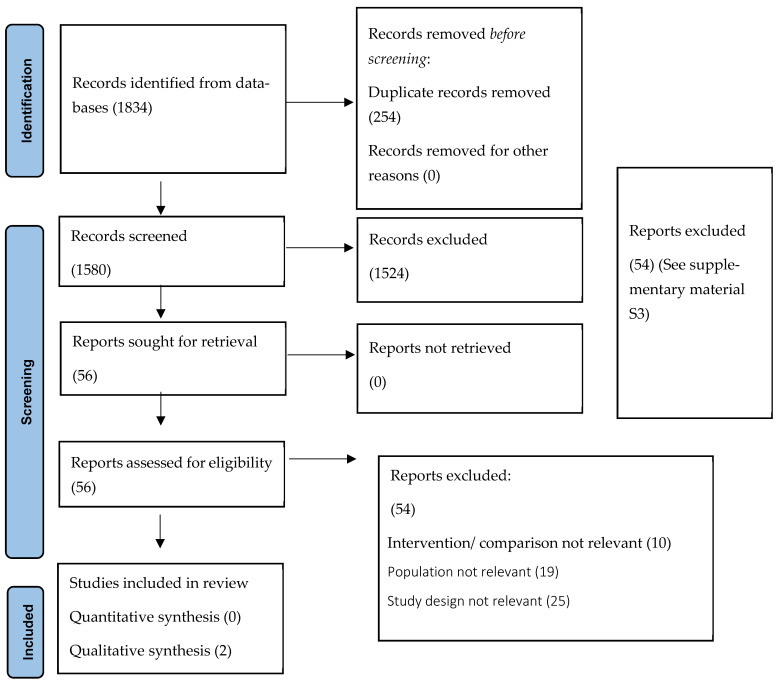
PRISMA flowchart.

A total of 1580 records were identified at the title and abstract stage. Abstract screening eliminated 1524 records, and the full text was obtained for 56 studies (see PRISMA diagram, Figure 2). No controlled experimental studies or observational comparative studies were identified for inclusion. However, we identified a case report and a case series, details of which are presented in Table 1. The study development group identified indirect evidence in accordance with the study criteria (see Table 2). This included seven primary research studies and five studies that were either narrative reviews, consensus meetings, or consensus guidelines. Given the novel term ‘gastrointestinal dystonia’, this indirect evidence made up the bulk of evidence identified.

### 3.1. Observational Non-Comparative Studies

Both the case report by Wahid and the case series by Hill describe the use of total parenteral nutrition for relief of symptoms of gastrointestinal dystonia. Wahid et al. provided in-hospital parenteral nutrition for a period of 5 months for a child with severe cerebral palsy who developed symptoms of bowel obstruction without mechanical cause, feed intolerance, and eventually complete intolerance of any enteral feed. This period of gut rest enabled the reintroduction of feeds over time, and after 12 months of in-patient stay, he was discharged home [8]

Cases like this pose the question of what are the underlying pathological processes involved in gastrointestinal dystonia. We know that children with severe neurological impairment can experience central pain from damage to the somatosensory system [22] Children exposed to multiple low-intensity nociceptive stimuli over a long period, from, for example, gastro-oesophageal reflux or constipation, may develop sensitisation of the central nervous system, amplifying any nociceptive signals from the gut due to chemical irritation and stretch. This sensitisation also occurs in peripheral neurons, resulting in visceral hyperalgesia, where normal gut movements/sensations are perceived as intense pain over a wide receptive field [23]. Due to impaired autonomic control of the gut, children with severe neurological impairment may have impaired gut motility [24]. This may be compounded by abnormal gut flora due to repeated hospital admissions and altered gut transit [25]. Children with damage to the brainstem are at risk of experiencing nausea and vomiting purely as a result of damage to the vomiting centres in the brainstem. In addition to these theorised contributors, it is important to remember that children with neurological impairment may lack the cognitive and emotional abilities to manage pain triggers, self-soothe, and rationalise complex symptom experiences. Displays of pain behaviours are complex in this group, and caregivers may inadvertently fail to respond to pain behaviours viewing them as normal for that child, or conversely become extremely anxious and distressed themselves, resulting in escalating anxiety for the child [26]. While a period of gut rest may have several positive effects in ameliorating nociceptive triggers and allowing emotional needs to be met, Wahid et al. did not describe any other interventions prior to the use of parenteral nutrition and initiation of palliative care that may have improved symptoms and feed tolerability.

Hill et al. describes a long-term home parenteral nutrition programme for five children with severe neurological impairment, reporting improvement in signs of distress but some persistent symptoms. Other analgesia was able to be reduced in the children receiving this intervention. One child died following a central line infection directly related to the use of parenteral nutrition. Four children died of their underlying condition, unrelated to the use of parenteral nutrition. The authors note that parenteral nutrition was required for a median of 5.5 yrs, which represents a significant burden of treatment for the child and family as well as an extensive home parenteral nutrition service. Again, there is no mention of interim measures or non-pharmacological/pharmacological approaches that may have been taken to improve symptoms [9].

Due to the study designs of these studies, low subject numbers, and differences between the interventions, the quality of the evidence supporting the use of parenteral nutrition for the management of symptoms relating to gastrointestinal dystonia is very low.

### 3.2. Indirect Primary Studies 

Seven primary studies were identified reporting interventions for children with severe neurological impairment and feed intolerance, pain behaviours associated with feeding, or severe gastrointestinal symptoms associated with neurological impairment. These studies, while published before the term gastrointestinal dystonia was defined, represent children who may have met the criteria for gastrointestinal dystonia if reviewed at the present time. Interventions identified to be beneficial in this group included reduction or withdrawal of enteral feed, use of parenteral nutrition, use of jejunal tube feeding at very slow rates titrated over time, use of extensively hydrolysed/elemental feeds initially, alimemazine for retching/vomiting, cyproheptadine for retching and feed intolerance, gabapentin for irritability and distress associated with feeds (three studies) pyridostigmine for lower gastrointestinal symptoms. Each of these studies reflects a certain aspect of management from unique perspectives. Mordekar et al., Manini et al.s and Merhar et al. all focus on interventions to improve gastrointestinal motility from the perspective of a gastroenterology team [10,12,16]. Antao describes a randomised cross-over study of fifteen children with neurological impairment after fundoplication, demonstrating that control of gasto-oesophageal reflux surgically solves one problem, only to reveal the underlying issues with hypersensitivity and an abnormal emetogenic reflex described above [11]. Hauer and Collins take a more holistic approach, taking into account the multiple factors involved in irritability in these children and demonstrating that use of gabapentinoids may help to manage symptoms and distress of any cause including gastrointestinal discomfort [13,14,15]. While each of these studies adds a valuable contribution to the broader picture, none manage to capture the entirety of the complex symptom experience facing these children and provide a coherent approach to management. Due to the study designs of these studies and low subject numbers, the quality of evidence supporting the use of these interventions would be classified as very low.

### 3.3. Reviews and Consensus Studies 

Five reviews or consensus studies were identified reporting recommendations based on expert opinion for a variety of patients, all with severe neurological impairment and gastrointestinal symptoms. These studies (with the exception of Hauer) again view this issue of GID from their own unique perspectives, taking into account a proportion of factors impacting children with GID. Hauer provides a more complete narrative review of the assessment and management of these children omitting the systematic review presented here [18]. The following interventions are reported as being effective in these studies:

Non-pharmacological:

- Use of the advanced care planning process to determine goals of care and support decision making;
- Use of home symptom management plans to guide caregivers in managing symptoms.
- Avoid over-feeding and calorie excess;
- Trial of 30% reduction in feed volume for 2–4 weeks;
- Bolus feeds of less than 15 mL/kg/feed, continuous feeds less than 8 mL/kg/h;
- Trial of continuous feeding/continuous nocturnal feeding with daytime boluses;
- Trial of hydrolysed or elemental formula;
- Trial of whey-based formula to improve gastric emptying;
- Avoidance of hyperosmolar feeds;
- Trial of blended diet;
- Gastrostomy tube venting to reduce distention;

Pharmacological interventions:

- Gabapentin or pregabalin or a tricyclic antidepressant for visceral hyperalgesia and central pain;
- Clonidine to reduce pain from gastric and colonic distention;
- Prokinetic drug to be tried prior to jejunal feeding;
- Consider trial of alimemazine in retching/vomiting;
- Consider trial of cyproheptadine in retching/vomiting;
- Consider trial of Neurokinin receptor antagonist (e.g., aprepitant) in retching/vomiting;
- Review medications to avoid polypharmacy where possible.

Other:

- Jejunal tube feeding trial as an alternative to fundoplication and gastrostomy feeding for children with severe gastro-oesophageal reflux with risk of aspiration.

Shortly following the completion of the searches for this review, Katz et al. published a case series of nine children with severe neurological impairment and intractable feed intolerance who were under a palliative care service in Australia [4]. While this study adds little in terms of additional interventions for suggested practice and study, the case series is notable for its attempt to capture the holistic picture affecting these children and outline approaches used for management across fields of gastroenterology, neurology, and palliative medicine. The authors note that the term ‘gastrointestinal dystonia’ appears as a diagnosis in several patient cases, but in addition to other terms including gastrointestinal dysfunction and intractable feed intolerance. Their inclusion criteria are very similar phenotypically to our above-stated definition of gastrointestinal dystonia in terms of symptom experience and burden of symptoms on quality of life: ‘A child was deemed to have intractable feeding intolerance if enteral feed related symptoms and/or suffering led to an inability to provide individual nutritional requirements and contributed to the overall deterioration or death of the child, for example, malnutrition leading to increased risk ofinfection and medical vulnerability, or death’ [4]. Of note, all children who were initially gastrostomy fed gained weight; however, as feed tolerance reduced, 7 out of 9 cases described had a trial of jejunal feeding. This again resulted in some initial weight gain, and children survived for a median of 16 months following this intervention. Parenteral nutrition was used as a tool in over half of the subjects, providing gut rest. It became clear in one of the two cases where it was used that continuation of parenteral nutrition served only to extend the dying process. After extensive discussion with the family and consultation with a clinical ethics service, it was discontinued. The authors rightly point out that where parenteral nutrition is used to sustain life in the context of gastrointestinal failure, its primary role in some cases may be to alter the mode of death (for example, to respiratory failure or seizure disorder) rather than extend good-quality life [4].

Another development has been the use of delta-9-tetrahydrocannabinol (Nabilone) for predominantly feed intolerance and upper gastrointestinal symptoms in children with severe neurological impairment. Brooks et al. in November 2024 published a case series of five patients with GID treated with Nabilone under guidance of a specialist centre [27]. All patients were on prokinetics, laxatives and in three of the four cases acid suppression. All were jejunely fed with three of the four patients receiving blended diet by gastrostomy in addition. One patient experienced excitation/euphoria and treatment was discontinued. For the remaining four patients, symptom severity measured using PedsQL V3 validated questionnaires improved following treatment and was sustained at 6 months. Clinically and statistically significant improvements were seen in total scores, scores for discomfort on feeding, and nausea and vomiting. Overall median weight remained static at 6 months when it would have been expected to de-cline without intervention [27]. Further studies are needed to establish the optimum dosing, true effectiveness and tolerability of nabilone but initial studies are suggest it may add another tool to the toolbox of GID management. 

### 3.4. Evidence to Decision Process

Certainty of the evidence of effectiveness in all cases was graded as very low in accordance with the GRADE process. Evidence was based on non-comparative observational studies with low patient numbers, expert opinion, or expert consensus. Due to the very low quality of evidence identified, the term ‘suggestions for practice and further study’ is used as it is not possible to make strongly substantiated recommendations on the basis of evidence identified. In the case of low quality evidence, discussion with patients and families is key to determining the benefits, harms, and overall best interests for each particular child. Following a whole-day consensus meeting using the ‘evidence to decision’ framework outlined by GRADE, reviewing the evidence by the study development group, combined with the expertise of the group and views of patient and parent representatives, it was agreed that the following suggestions for practice be made. All practice points were agreed upon unanimously, and discussion continued until consensus on wording was agreed. Where ‘suggestions for practice and study’ are made based on study group consensus, this is made clear. Where suggestions for practice and study are based on evidence, this is also made clear below.

## 4. Suggestions for Practice and Further Study

### 4.1. General Approach to Management

- An overall clinical lead should be identified to coordinate the multiple teams involved in managing a patient with gastrointestinal dystonia [8]. (consensus view of study development group) Gastrointestinal dystonia is a multidimensional diagnosis with several teams involved in management. Establishing clear leadership and regular multidisciplinary team meetings is the mainstay of management of gastrointestinal dystonia [8].
- Agree goals of care with the family and multidisciplinary team involving the nutrition team, neurology, and palliative care at diagnosis and points of deterioration [8]. (consensus view of study development group).
- An advance care planning process should be started at diagnosis of gastrointestinal dystonia with patients, their caregivers, and the wider multidisciplinary team [2]. (very low-quality evidence). Using an advance care planning process to support goal setting and decision-making is integral to ensuring clear, goal-driven management and avoiding harmful interventions or futile treatment trials. Advance care planning involves a series of discussions between the child, caregivers, and the medical team to gain a joint understanding of the condition, wishes, hopes, fears, and uncertainties, and create plans in the event of deteriorating health. It is not unusual for a number of plans to be made to cater for a variety of scenarios as the child’s condition progresses. Topics for discussion include the following:
  - Ensuring a shared understanding of the child’s condition, natural history, and expected disease trajectory.
  - Understanding with wishes, goals, fears, hopes, and anxieties of the child and family as treatment progresses.
  - Agreeing management of distressing symptoms and developing an associated ‘symptom management plan’ to guide caregivers in managing symptoms in the community.
  - Agreeing management of episodes of acute deterioration in health, including assessment at home or in hospital and escalation of life-sustaining treatment as appropriate.
  - Agreeing management of gradual deterioration should it become clearer that the child will not survive.
  - Considering wishes of the child and caregivers with regard to end of life care and discussing changes to the body, location of care, adjustments made to medications and treatment, and post death wishes.
  - Discussing support needs of the child and family [28].

Plans are shared with all professionals involved, including school, respite care, and emergency care organisations, to ensure, as far as possible, that the wishes of the child and family are the centre of care and best interest decisions are able to be made more easily during any deterioration in health condition.

- Clear symptom management plans should be developed with caregivers to guide the assessment and management of distressing symptoms in the community and who to call for advice or support if required (consensus view of the study development group). Symptom management plans may include the following:
  ▪ Clear description of the symptom, and how it presents for that specific child;
  ▪ Initial non-pharmacological approaches to managing the symptom in a safe space;
  ▪ Pharmacological management of the acute symptom including dose frequency of any medications given;
  ▪ Next steps if initial steps are ineffective;
  ▪ Who to call for advice or support if symptoms persist despite the above measures [28].

### 4.2. Initial Management of Gastrointestinal Dystonia

- Fully assess for sources of pain and distress other than gastrointestinal dystonia (consensus view of study development group) [26,29].
- Ensure optimal management of gastro-oesophageal reflux, spasticity, muscular dystonia, secretions, and constipation prior to treatment directed at gastrointestinal dystonia (consensus view of study development group) [21]. Children with severe neurological impairment often have multiple sources of pain and distress [26,29]. These should be fully assessed and excluded or treatment optimised prior to treatment directed specifically at gastrointestinal dystonia (consensus view of study development group). If hip or spine pain, spasticity, or gastroesophageal reflux disease is poorly controlled, management of gastrointestinal dystonia will likely be ineffective as significant triggers for pain and distress remain unaddressed.
- Avoid polypharmacy where possible, reducing medication burden where possible at diagnosis of gastrointestinal dystonia (very low-quality evidence) [18,20].
- Where possible, medications known to impact gastrointestinal motility, such as anticholinergics (trihexyphenidyl, tricyclic antidepressants, hyoscine hydrobromide/butylbromide), specific opioids (morphine, diamorphine, oxycodone, etc.), and serotonin receptor antagonists (ondansetron/granisetron) should be weaned or removed if possible (consensus view of study development group).
- Avoid over-estimating fluid and calorie requirements based on weight, length, weight z-scores, and BMI alone, as this may lead to excess feed being administered and impair tolerance. Assessment by a dietician with experience working with children with severe neurological impairment is advised (very low-quality evidence) [21].
- Optimise feed regimen before starting medications for gastrointestinal dystonia (very low-quality evidence) [18,20,21].
- Modify feed regimen and composition prior to use of medications for gastrointestinal dystonia (very low-quality evidence) [18,20,21].
- Consider reducing overall feed quantity per day to a point where tolerance is achieved to manage symptoms (very low-quality evidence) [18].
- Consider use of a blenderised diet prior to post-pyloric feeding for managing symptoms where appropriate (very low-quality evidence) [19,30].

A realistic assessment of weight gain should be made, as patients with gastrointestinal dystonia may not follow normal growth percentiles. This is vital to provide optimal nutrition and avoid overfeeding [21] (very low-quality evidence). Calorie requirement in children with severe neurological impairment is commonly overestimated, and overfeeding is a common contributor to feed intolerance [18,20]. Algorithms for calculating nutritional requirements can only provide an estimate; be prepared to modify feed intake in light of actual growth and other nutritional markers [20]. Before initiating medications to manage distressing symptoms, management should include optimisation of feed volume (e.g., a trial of a 30% reduction for two weeks or periods of permissive undernutrition) and composition (lower osmolality feed or hydrolysed formulas) (very low-quality evidence) [18]. Some children benefit from a blended diet delivered by gastrostomy to manage upper gastrointestinal symptoms and pain/distress during feeds, and this should be trialed early following dietetic assessment of the child and family circumstances [17] (very low-quality evidence).

- Develop a plan to manage anxiety and agitation alongside symptoms of gastrointestinal dystonia. This may include use of clonidine or a short-acting benzodiazepine (such as midazolam) enterally or oral-transmucosally (if faster onset of action is required) alongside psychological interventions and management of the environment and caregiver anxiety/distress (consensus view of the study development group).
- Children and young people with severe neurological impairment are especially sensitive to environmental factors (noise, lighting, and emotion) and the emotions of their primary caregivers during periods of symptom occurrence. Ensuring a clear plan for psychosocial and spiritual factors for the patient and caregivers is an essential part of management. This requires a multidisciplinary approach [26] (consensus view of the study development group).

### 4.3. Pharmacological Management of Pain

The following suggestions for practice aim to reduce symptom generation through reduction in gastrointestinal distention and support of regular peristalsis and gastric emptying, as well as managing visceral hyperalgesia. The management of pain-related anxiety and emotional distress is also considered.

- Alongside simple analgesics such as paracetamol enterally or rectally, consider a gabapentinoid first line for pain in gastrointestinal dystonia (very low-quality evidence) [13,14,15]. Gabapentin has been demonstrated to be effective for managing pain in patients with severe neurological impairment and is recommended as first-line for central pain, visceral hyperalgesia, and pain of unknown origin [15]. It is well tolerated and has been reported to improve feed tolerance. The impact of opioids on motility precludes their use as long-acting agents in the management of early gastrointestinal dystonia (consensus view of the study development group).
- Consider the addition of clonidine enterally if pain symptoms persist [31]. Clonidine acts as an antineuropathic agent to reduce visceral hypersensitivity, as well as an anxiolytic. Although relative bradycardia and hypotension have been noted with its use in a variety of indications, there is no evidence that these lead to clinical deterioration when national prescribing guidelines are followed [31]. Clonidine may be used enterally, transmucosally, transdermally, or intravenously (consensus view of the study development group).
- Opioids should generally be avoided as first-line medications due to the impact on gastrointestinal motility. However, a variety of synthetic opioids known to be less constipating may be considered. These include buprenorphine and fentanyl (both available transdermally). Should morphine/oxycodone or other more constipating opioids be used, consider the concurrent use of a peripherally acting mu-opioid receptor antagonist to reduce the impact of constipation (consensus view of the study development group).
- Third line antineuropathic agents may be used under specialist advice for the management of pain. These include ketamine enterally or parenterally or methadone enterally or parenterally [29]. The effectiveness of newer agents for neuropathic pain, such as oxycarbazepine and lacosamide, in GID, is yet to be established (consensus view of the study development group). Seek specialist advice.

### 4.4. Pharmacological Management of Retching and Vomiting

- Nausea, vomiting, and retching may be as a result in the following factors:
  - Damage to brain areas involved in the vomiting reflex and emesis generation.
  - Gastrointestinal dysmotility, including delayed gastric emptying.
  - Autonomic dysfunction.
  - Visceral hypersensitivity of the upper gastrointestinal tract.
  - Gastro-oesophageal reflux, cough, and secretion clearance due to impaired swallowing.
  - Emetogenic medications.
  - Pain.
  - Psychological factors.
- Consider a prokinetic first-line for management of upper gastrointestinal symptoms (very low-quality evidence) [20].
- Cyproheptidine hydrochloride or alimemazine should be considered as second line agents where available (very low-quality evidence) [11,12].
- Management of visceral hypersensitivity and pain may be required for resistant symptoms of retching/vomiting (very low-quality evidence) [18]. Autonomic dysregulation, visceral hyperalgesia, and impaired gut motility underlie much of the distress related to the upper gastrointestinal tract in children with severe neurological impairment.
- Antiemetics acting on the chemoreceptor trigger zone and vomiting centre of the brain may be used with caution, for example, levomepromazine and neurokinin-1 receptor antagonists (consensus view of the study development group). Avoid antiemetics with high anticholinergic activity (hyoscine hydrobromide/butylbromide/cyclizine) or those otherwise negatively impacting on gut motility (serotonin receptor antagonists ondansetron/granisetron) (consensus view of the study development group).
- Once the above approaches have been trialed if symptoms persist consider a trial of delta-9-tetrahydrocannabinol (Nabilone) under specialist advice.20 (very low quality evidence) [27].

### 4.5. Pharmacological Management of Lower Gastrointestinal Symptoms

CYP with GID may present with prolonged periods of constipation, pain on defecation or passing flatus, and may present with symptoms similar to those seen in intestinal obstruction (distention, pain, and bowels not opening). The following factors contribute to these symptoms:

- History of chronic constipation leading to alterations in bowel compliance;
- Upper motor neurone damage leading to sphincter dysfunction;
- Autonomic dysfunction;
- Visceral hyperalgesia;
- Intestinal dysmotility;
- Medications leading to impaired peristalsis.

▪ With regard to constipation, painful defecation, and intermittent intestinal obstruction symptoms in children with gastrointestinal dystonia, national guidance for the management of constipation in children should be followed in the first instance [21,32] (consensus view of the study development group). The caveat to this is the impact of upper motor neuron damage on sphincter activity in the gut in children with severe neurological impairment. Due to this, active bowel management using suppositories and enemas may be needed alongside enteral management [21] (very low-quality evidence).
▪ Use of regular suppositories and/or enemas early in management to help regulate bowel movements and reduce episodes of distressing unpredictable bowel opening/flatus (very low-quality evidence) [21].
▪ Consider use of prucalopride in older children with resistant obstructive symptoms (consensus view of the study development group) [33,34]. Prucalopride is a 5HT4 receptor agonist with mostly lower GI prokinetic effects. For older children only under specialist advice.
▪ There is insufficient evidence or experience to recommend the use of linaclotide for this group of patients. Linaclotide is a guanylate cyclase-c agonist, increasing secretion of ions and water into the GI tract. Seek specialist advice (consensus view of the study development group).

### 4.6. Bloating, Flatulence

- Management of bloating usually improves as a result of optimisation of the symptoms discussed above and alterations to enteral feed described previously. Probiotics may be considered as a trial for 8–12 weeks while enteral feed is tolerated, and a course of treatment for small intestinal bacterial overgrowth may be of benefit (consensus view of the study development group). Peppermint tea or oil can be of benefit, according to some families, though evidence is lacking. Use of regular suppositories as above for managing constipation may improve the regulation of flatulence causing discomfort [21].

### 4.7. Agitation and Anxiety

- Management of agitation and anxiety is vital, as these play a key role in distress expressed by children with GID (consensus view of the study development group).
- Management of anxiety should be included in symptom management plans for pain or distress episodes. A combination of comfort measures a rapid-onset (fentanyl) or immediate-release analgesic alternating with a rapid-acting benzodiazepine (e.g., buccal midazolam) may be required to manage both pain and anxiety components of distress episodes. Seek specialist advice for more detailed management (consensus view of the study development group).

### 4.8. Clinically Assisted Hydration and Nutrition

- Decisions around the use of clinically assisted nutrition or hydration are challenging and complex. Cesacion of feeds and initiation of parental nutrition may reduce symptoms related to GID.(very low quality of evidence) [8,9]. Parenteral nutrition may be started acutely during episodes of illness or acute severe distress associated with feed intolerance. In these instances there may not be time to consider fully the implications if treatment is continued longer term. In addition, for the child with complete feed intolerance, continuing on parenteral nutrition may seem to be the only option facing families and professionals when the alternative of palliation is unbearable. The parent representative on our study group emphasised the value of parenteral nutrition in facilitating periods of good quality life, using the example of her son, who survived for a number of years with total parenteral nutrition at home. This view is consistent with the findings from Rapoport et al., who reported that parents’ perception that their child’s quality of life was poor was an important prerequisite to considering withdrawal of hydration and nutrition [35]. However, our parent was clear that the burdens of long term parenteral nutrition were significant, including central line care and infection risk, liver complications, and ultimately witnessing her child’s neurological decline continuing despite the nutritional support. The study development group agreed that the correct approach should be focusing on the individual needs, wishes, and values of the child and family, considering what can be practically provided given local resources, and with reference to robust ethical frameworks and advice. Support for all involved is vital, including medical and nursing teams involved in decision making (consensus view of the study development group).

Rapoport et al., who interviewed bereaved parents who had taken the decision with professionals to withdraw parenteral nutrition, emphasise that the following are important to parents and caregivers with respect to these decisions:

- Parents needed to feel ready for discussions. Parents felt ready for discussions when they perceived their child’s quality of life as poor. They needed to recognise their child as on a trajectory towards dying.
- The child’s physician should communicate the discussions and ethical justification to families.
- Parents valued time to consider the decisions.
- Parents valued physicians sharing their prior experiences of similar cases with them.
- Parents valued professionals being clear and confident in the views of the medical team when recommending withdrawal of hydration and nutrition (the importance of a clinical lead and strong multidisciplinary team engagement with regular meetings).
- Parents valued the ability to give their child some form of comfort feeding if tolerated [35].

▪ Any trial of assisted hydration or nutrition, especially parenteral nutrition for gastrointestinal dystonia, should be started with agreement on goals of care and timescale of the trial, considering benefits and harms of treatment, and implications of provision for quality of life and location of care [4,8,9] (very low-quality evidence). Decisions regarding clinically assisted hydration and nutrition are particularly pertinent in gastrointestinal dystonia. Clinically assisted nutrition includes intravenous feeding and feeding by nasogastric tube and by percutaneous endoscopic gastrostomy (PEG) and radiologically inserted gastrostomy (RIG) feeding tubes. Clinically assisted hydration can also be provided by intravenous or subcutaneous infusion of fluids [36]. In line with guidance from the UK General Medical Council and Royal College of Paediatrics and Child Health, artificial hydration and nutrition may be initiated or withdrawn on the basis of a multidisciplinary assessment of best interests with a clear timeframe and review point [36,37,38].
▪ Nutrition and hydration interventions should be assessed separately [36].
▪ The advance care planning process should be underway with agreed goals of care before starting a trial of parenteral nutrition, or if parenteral nutrition is started during an acute illness, as soon as practically possible (consensus view of the study development group).
▪ Starting parenteral nutrition is not appropriate where it does not meet agreed goals of care or where the overall burdens outweigh the benefits. This may include the burden to the child and family of remaining in the hospital (consensus view of the study development group).
▪ In a situation of disagreement between caregivers and professionals, hospital legal teams will be able to advise on next steps if a second opinion, clinical ethics review, or use of mediation services does not lead to an agreed path forward.(consensus view of the study development group)

### 4.9. End-of-Life Care

Sadly, some children and young people with GID are unable to tolerate food and die as a result of nutritional failure. At the end of life, reduced food and fluid requirements are a natural part of the dying process. The desire for hydration and nutrition also diminishes during the dying phase. This can make assessment of benefits and harms of continuing to provide assisted nutrition and hydration challenging for families and professionals.

- In the sad situation where it is clear that a patient is likely to die in days or short weeks, it is important that discussions are had pre-emptively with the caregivers specifically about hydration and nutrition at the end of life (consensus view of the study development group). The reduction or withdrawal of artificial hydration and nutrition is highly emotive for families and professionals.
- As the child’s condition deteriorates towards the end of life, goals of care shift more completely towards comfort and control of distressing symptoms and away from nutrition and growth. It may be appropriate to slow down gastrointestinal motility as obstructive symptoms progress. This approach would usually involve discontinuing prokinetic agents, placing gastric feeding tubes on drainage if still in place, and using anticholinergic and antisecretory medications (consensus view of the study development group).
- Offer advice and support if caregivers wish to persist with enteral feeding and it brings pleasure to the patient, even if considered a risk or ineffective, so long as this does not cause the child distress (consensus view of the study development group).

### 4.10. Future Research

Gastrointestinal dystonia is a recently defined clinical diagnosis in an emerging population. More research is required to understand the population of patients with the condition, how the various presentations interact, and support identification of clusters of symptoms in practice. While the various pharmacological approaches recommended are established treatments within paediatrics, there is no experimental evidence for their use in this population, which needs to be a focus of future research. The impact of aligning the advance care planning process used routinely in palliative care with a diagnosis of gastrointestinal dystonia again needs to be evaluated with patient- and caregiver-reported feedback measures developed. This is particularly important in relation to the use of artificial hydration and nutrition in this group.

## 5. Limitations

The results of this study are limited by the lack of experimental trial evidence for the use of specific interventions specifically in a population of children diagnosed with GID. The heterogenous nature of the condition and recently defined diagnosis lead to conditional and weak recommendations in this group of patients.

## 6. Conclusions

Gastrointestinal dystonia is a highly distressing, holistically complex, and heterogeneous condition. Recommendations generated from this study demonstrate that such conditions are not beyond the reach of a pragmatic and rational approach to initial management. Many of the interventions described can be undertaken by those working in local hospital and community teams anywhere in the world, supported by specialist centres where necessary. As children with life-limiting conditions live longer due to advanced respiratory and neurological techniques, new and emerging multifaceted conditions like gastrointestinal dystonia will need to be addressed by large multidisciplinary teams within paediatric and adult services alike.

## Figures and Tables

**Table 1:** Summary of observational non-comparative studies.

Study Details	Participants	Interventions	Main Results
Wahid 2017 [8] UK Case report Hospital setting Dates not reported.	13-year-old boy with life limiting condition. Cerebral Palsy (CP), epilepsy, severe learning difficulties, and autistic features. Palliative child with intestinal failure.	Central venous catheter (CVC) and total parenteral nutrition (PN). Nasogastric (NG) feeds were not tolerated and nasojejunal (NJ) feeding was also unsuccessful.	TPN was provided post-operatively and continued for five months. During this period the child did not tolerate NJ feeds. Severe gut dysmotility was confirmed throughout the remaining gut. Slow introduction of comfort feeds via NG and mouth. This gradually improved as the child started to tolerate more volume and reached full enteral feeds by 3 months. TPN may be indicated for a period of gastrointestinal rest prior to reintroduction of feeds in children with gastrointestinal failure.
Hill 2021 [9] Case series 2009–2019.	Six children with intestinal failure and severe neurological impairment.	Home parenteral nutrition programme.	Survival time of 3–7.8 years (median 5.5 years). “Improvement in signs, but not symptoms, reduced analgesia” “no more complications than expected”. Deaths = 4; (3 deaths unrelated to PN and 1 with central line associated bloodstream infection).

**Table 2:** Summary of indirect studies identified.

Indirect Evidence from Primary Studies					
Study ID	Methods	Population	Intervention(s)	Study results	Notes
Paper 1: “Feed-induced dystonias in children with severe central nervous system disorders.” S Mordekar, M Velayundhan, D Campbell. United Kingdom 2012–2017 [10]	Retrospective case series.	12 children age 5 months–15.8 yrs (mean 9.5 yrs) patients presenting with status dystonicus with evidence of feed-induced dystonic spasms. Patient with gastro-oesophageal reflux disease (GORD) or Sandifer syndrome excluded.	Withholding feeds and use of TPN to manage symptoms. Octreotide intravenously in 2 cases	Withholding feeds led to a resolution of symptoms. Restarting even 5 mL/h via gastric or jejunal tube using extensively hydrolysed/elemental or electrolyte solution led to the return of dystonia. Patients with feeds withheld experienced resolution of symptoms.	All taking anti-dystonic medications. All had pH studies, gastroscopy, and biopsies with no evidence of GORD or oesophagitis. Eight had fundoplication (confirmed to be intact).
Paper 2: “Effectiveness of alimemazine in controlling retching after Nissen fundoplication” Antao et al. United Kingdom December 2002–2003. [11]	Prospective double blind randomised crossover placebo-controlled study. Exclusion criteria: hepatic or renal impairment, hypothyroidism.	15 subjects, 12 enrolled with completed diaries of retching episodes. Age 8–180 months (median 36). Patients with neurological impairment. Post-nissen fundoplication for GORD. All gastrostomy fed.	One week alimemazine, one week placebo with crossover. Alimemazine 0.25 mg/kg three times daily (max 2.5 mg per dose).	Mean number of retching episodes with alimemazine significantly reduced compared with placebo. No adverse effects of Alimemazine reported. One subject discontinued due to drowsiness. Concluded Alimemazine safe and effective for retching post-nissen fundoplication.	Recommended dosing 0.25 mg/kg three times daily.
Paper 3: “A retrospective review of Cyproheptadine for feeding intolerance in children less than three years of age: effects and side effects.” Merhar et al. United States 2011–2015 [12]	Retrospective chart review of children under 3yrs of age seen in neonatal follow up clinic prescribed Cyproheptadine for feed intolerance (fullness or discomfort with feeds, retching and/or abdominal distention or vomiting).	39 children under 3 yrs. Graduates from the neonatal unit(18 with prematurity (46%) 29 with perinatal brain injury (74%)). 61% artificially tube fed, 59% by gastrostomy. 23.5% had nissen fundoplication. Most remained on an ‘acid blocker’.	Cyproheptadinemean starting dose 0.23 mg/kg/d three times daily (range 0.07–0.83 mg/kg/d).	Side effects noted: 25.6% mild sleepiness, 10.2% constipation, 7.7% behavioural tantrums increasing. 5.1%–discontinued medication. Sleepiness and constipation resolved with dose reduction. Statistically significant weight increase on treatment. Outcomes; 66.7% symptom resolution, 28.2% some improvement in symptoms, 84.6% improved vomiting.	Recommended dosing 0.5–1 mg/kg/day in three divided doses for patients <1 yr. 1–2 mg/kg/day in three divided doses for patients in older children.
Paper 4: “Gabapentin for the treatment of pain manifestations in children with severe neurological impairment: a single centre retrospective review” Collins et al. Ireland 2019 [13]	Single centre retrospective chart review.	42 patients attending joint gastroenterology and palliative care services with gastrointestinal cause of pain and distress. 3–63 m age.	Gabapentin for central pain and visceral hyperalgesia. Pregabalin as second line option if gabapentin response inadequate or side effects.	Response to gabapentin good or very good overall in 60% patients. Worse in 2%. 71% noted improvement in irritability. 40% noted improvement in pain (reduced analgesia requirement) 55% no improvement, 5% undocumented. Side effects: None in 74%, reducing effectiveness in 10% (taking 60 mg/kg/d), lethargy in 7%, alopecia, twitching, vomiting, abnormal liver function tests also reported. Discontinued in 36% with 80% of these switched to pregabalin.	Not clear what criteria was used for good or very good effect apart from notes report. No objective measures used. Also included patients with irritability of unknown origin as well as those with gastrointestinal symptoms.
Paper 5 “Gabapentin Successfully Manages Chronic Unexplained Irritability in Children With Severe Neurologic Impairment” Hauer JM, Wical BS, Charnas L. 2006 United states 2006. [14]	Retrospective single centre case series	Nine patients age 9 m—22 yrs with severe neurological impairment and irritability. Population had feed intolerance and irritability associated with feeds, defecation and flatus. Suspected due to visceral hypersensitivity. Six out of nine gastrostomy tube fed, seventh scheduled for gastrostomy.	Gabapentin	All cases had upper gastrointestinal endoscopy several had PH studies with no significant pathology. All tried acid suppression with no improvement. Eight out of nine were treated successfully for constipation. Marked improvement with gabapentin trial. 5 mg/kg/dose once daily at night, increased every 3–7 days final doses 15–35 mg/kg/day in three divided doses. Improved irritability, crying, feed tolerance, sleep, and improved responsiveness.	One child who discontinued treatment had a good response to amitriptyline. No objective data to measure ‘marked improvement’.
Paper 6 “Gabapentin for Management of Recurrent Pain in 22 Nonverbal Children with Severe Neurological Impairment: A Retrospective Analysis” Hauer JM, Solodiuk JC. 2015 United States 2011–2014 [15]	Single Centre Retrospective study case note review.	22 patients with severe neurological impairment in long term paediatric care facility with recurrent pain behaviours recorded on Individualised numeric rating scale (INRS). 64% had gastrointestinal symptoms. Mean age 11.4 yrs (range not given but up to 27 yrs).	Gabapentin 5–6 mg/kg/day increased every 2–3 days to maximum 72 mg/kg/d or until symptoms improved. (Mean dose required 43 mg/kg/day.) All on medication for gastric acid suppression.	Significant benefit (>50% reduction in frequency and severity) in recurrent pain behaviours in 21 patients (95%). Some improvement in dystonia also noted. Some had a decrease in vomiting, two who were jejunely fed were able to return to gastric feeding, weight gain noted in some.’ No significant side effects—mild transient sedation in a few.	Criteria for benefit used was if two or more bedside nurses reported the patient to have greater than 50% benefit along with decrease in frequency and severity of episodes irritability. (Pain frequency determined by the use of ‘as required’ analgesics.)
Paper 7 “Application of Pyridostigmine in paediatric gastrointestinal motility disorders: A case series.” Manini ML, Camilleri M, Grothe R, Lorenzo CD. United States 2017 [16]	Retrospective case series.	Heterogeneous population. Case series with one relevant case of child with severe neurological impairment and feed intolerance 7 yrs old.	Distention and poor motility despite laxatives. Pyridostigmine 10 mg four times daily (4 mg/kg/day) reduced to 2 mg/kg/day maintenance.	Significant increase in frequency of bowel opening (from every 5 days to daily). Vomiting and abdominal distention improved. Feed tolerated at 50 mL/h over 20 h daily. Weight gain continued. Stopping pyridostigmine lead to symptom recurrence.	
**Reviews and Other Non-Primary Sources**					
Study ID	Methods	Population	Intervention(s)	Main conclusions/Recommendations	
Review 1. “Blended foods for tube-fed children: a safe and realistic option? A rapid review of the evidence” Coad et al. 2016 United Kingdom August—December 2014 [17]	Rapid review of literature using systematic principals. PubMed, Medline, Cinahl, PsychINFO, Google Scholar.	Children and adults using blended diet as alternative feed.	Blended diet to replace commercial formula for feeds.	Significant social benefit of blended diet and parent satisfaction. Improved tolerance of greater feed volume, reduction in pain, reflux, and constipation with its use.	
Review 2. “Feeding intolerance in Children with Severe Impairment of the Central Nervous System.” Hauer J United States 2017 [18]	Narrative literature review and guideline.	Children with severe neurological impairment and feed intolerance.	Multiple	Multiple, including: Importance of home care plans for parents to follow to manage symptoms. Importance of advance care planning to manage goals of care and expectations of care. Consider 30% reduction in feed volume with monitoring of weight and symptoms to assess benefit 2–4 weeks. Bolus feeds should be <15 mL/kg/feed, continuous rate <8 mL/kg/h. Gastrostomy tube venting to reduce GI distension. Gabapentin pregabalin and tricyclic antidepressant trial for visceral hyperalgesia and central pain. Clonidine for pain perception during gastric and colonic distention. Cyproheptadine to improve feed tolerance, decrease emesis and retching post-fundoplication.	
Review 3: “The use of jejunal tube feeding (JTF) in children: A position paper by the Gastroenterology and Nutrition Committees of the ESPGHAN 2019.” Broekaert et al. 2019 Pan European Literature review until 2018. [19]	Systematic literature review and consensus meeting. Literature review Medline 1980–2015. PubMed and Cochrane. Evidence categorised according to GRADE. Consensus vote on recommendations.	Patients for whom jejunostomy tube feeding is a consideration.	Jejeunostomy tube insertion and management.	Decision to place jejunostomy tube should be multidisciplinary. A trial of continuous gastric feeding with hydrolysed or elemental formula should be given prior to JTF. Consider a trial of at least 1 prokinetic drug to promote oral or gastric feeding before instituting jejunal feeding. In children with severe neurological impairment JTF should be considered as an alternative to fundoplication and gastrostomy feeding where the child has severe GORD with risk of aspiration.	
Review 4: “Does retching matter? Reviewing the evidence—Physiology and forces.” Richards C. A. United Kingdom. 2019 [20]	Narrative literature review	Children with severe neurological impairment post antireflux surgery.	Management of retching and vomiting in post-reflux surgery patients.	Trial of whey-based feed leading to improved gastric emptying Predigested formula trial Avoid hyperosmolar feeds Consider blenderised feeds Avoid overfeeding and calorie excess Smaller more frequent boluses Continuous gastric feeds Consider jejunal feeding +/− gastric drainage Consider prokinetic (Domperidone and metoclopramide) trial Consider trial of alimemazine Consider trial of Cyproheptadine Considrer trial of Seratonin 5HT3 antagonist Consider Levomepromazine. Consider trial of Neurokinin receptor antagonist Reduce/review polypharmacy especially when many drugs present.	
Review 5 “European Society for Paediatric Gastroenterology, Hepatology and Nutrition Guidelines for the Evaluation and Treatment of Gastrointestinal and Nutritional Complications in Children With Neurological Impairment” Romano et al. Pan-european. 2017 [21]	Systematic literature review and consensus meeting. Literature review Medline 1980–2015. Consensus vote on recommendations.	Children with neurological impairment. Children with cerebral palsy referred to as the major subgroup of purpose of this document.	Management of nutrition and gastrointestinal problems in children with neurological impairment.	Optimise nutrition and avoid overhydration. Trials of feed thickener and whey-based formulas should be considered. Use of prokinetics should be reserved for uncontrolled GORD due to weak efficacy and side effects. Regular re-evaluation of efficacy. Constipation: Ensure adequate fibre and fluid intake. Initial treatment with disimpaction and osmotic maintenance agents. Macrogol and paraffin should be used in caution with those at high risk of aspiration. Enemas may be required to support faecal disimpaction alongside osmotic agents. Standard 1 kcal/mL polymeric age-appropriate formula is the appropriate first feed option For poor volume tolerance consider high energy density (1.5 kcal/mL) formula containing fibre. Blenderised diets can be used but with caution due to concerns about nutritional adequacy and safety. A combination of continuous nocturnal feeds and daytime bolus feeds should be considered in those with high caloric needs or with poor tolerance to volume.	

## Data Availability

Not applicable.

## Supplementary Materials

The following supporting information can be downloaded at: https://www.mdpi.com/article/10.3390/children12101359/s1. S1: Search strategy. S2: PRISMA checklist. S3: Table of excluded studies.
