# Supplementary material for: Gastrointestinal Dystonia in Children and Young People with Severe Neurological Impairment & Palliative Care Needs: A Systematic Review
Source: Children (Basel). 2025 Oct 9;12(10):1359. doi: 10.3390/children12101359 (PMC12562431; doi:10.3390/children12101359)
Supplement: Supplementary file 1 [file children-12-01359-s001.zip › S1 Search Strategy.pdf]

S1. Search strategy MEDLINE

(Ovid) Search Strategy

1. ADOLESCENT/ or MINORS/

2. (adolescen$ or teen$ or youth$ or young or juvenile? or minors or highschool$).mp,jw,nw.

3. exp CHILD/

4. (child$ or schoolchild$ or "school age" or "school aged" or preschool$ or pre-school* or toddler$ or kid? or kindergar$ or boy? or girl?).mp,jw,nw.

5. exp INFANT/

6. (infan$ or neonat$ or newborn$ or baby or babies).mp,jw,nw.

7. exp PEDIATRICS/ or exp PUBERTY/

8. (p?ediatric$ or pubert$ or prepubert$ or pubescen$ or prepubescen$).mp,jw,nw.

9. or/1-8

10. TERMINALLY ILL/

11. ((terminal$ or final or advance$ or incurable or life limit$) adj3 (ill$ or disease$ or condition$)).mp.

12. dying.mp.

13. (end adj3 life).mp.

14. ((approach$ or close$ or near$ or imminent$ or impending) adj3 death).mp.

15. (Body adj2 (shut? down or shutting down or deteriorat$)).mp.

16. (deathbed? or death bed? or passing away or passing on or expiring or expiration or syringe driver*).mp.

17. ((last or final) adj1 (hour$ or days$ or minute$)).mp.

18. (last year of life or LYOL or life$ end).mp.

19. (advance$ stage? or final stage? or end stage? or last stage? or late stage? or terminal stage?).mp.

20. ((advanced or late or last or end or final or terminal) adj phase$).mp.

21. RESUSCITATION ORDERS/

22. (resuscitat$ adj3 (policies or policy or order? or decision? or withhold$)).mp.

23. ADVANCE DIRECTIVES/

24. advance? directive?.mp.

25. LIVING WILLS/

26. living will?.mp.

27. TERMINAL CARE/

28. (terminal$ adj3 (care$ or caring)).mp.

29. PALLIATIVE CARE/

30. palliat$.mp.

31. HOSPICE CARE/

32. hospice?.mp.

33. or/10-32

34. exp Intestinal Obstruction/

35. exp Constipation/

36. (ileus or constipat*).mp.

37. ((gut or gastrointestin* or gastro-intestin*or intestin* or bowel or colon* or duoden* or jejun* or ileum or ileal) adj3 (obstruct* or fail* or dysmotilit*)).mp.

38. 34 or 35 or 36 or 37

39. 9 and 33 and 38

40. 33 and 38

41. limit 40 to ("all infant (birth to 23 months)" or "all child (0 to 18 years)" or "newborn infant (birth to 1 month)" or "infant (1 to 23 months)" or "preschool child (2 to 5 years)" or "child (6 to 12 years)" or "adolescent (13 to 18 years)")

42. 39 or 41

43. exp animals/ not humans/

44. 42 not 43

45. (comment or historical article or news).pt.

46. 44 not 45
