# Supplementary material for: Gastrointestinal Dystonia in Children and Young People with Severe Neurological Impairment & Palliative Care Needs: A Systematic Review
Source: Children (Basel). 2025 Oct 9;12(10):1359. doi: 10.3390/children12101359 (PMC12562431; doi:10.3390/children12101359)
Supplement: Supplementary file 1 [file children-12-01359-s001.zip › S3 Table of Excluded Studies.pdf]

S3. Excluded studies

Refid	Bibliography	Reason for exclusion
2	2010. Other complementary therapies Focus on alternative and complementary therapies, 15(2): 175.	Study design not relevant
30	Agarwal T, Aboumarzouk O. Shariff U. Antakia R. Nelson R. L. 2010. Cisapride for intestinal constipation Colorectal disease, 12(#issue#): 38.	Population not relevant
55	Almeida, P. S., Penna, F. J. 2000. Chronic intestinal Pseudo-obstruction in childhood International Pediatrics, 15(2): 79-86.	Intervention/ comparison not relevant
62	Amin, Seema, Dickerman, Mindy, Miller, Elissa, Levy, Carly 2021. Transdermal buprenorphine in children with complex chronicconditions: A case series Pediatrics, 147(3): 550.	Population not relevant
67	Anonymous 1980. Constipation Nursing, #volume#(17): 751-5.	Study design not relevant
82	Athavale, Akshay, Athavale, Tegan, Roberts, Darren M. 2020. Antiemetic drugs: What to prescribe and when Australian Prescriber, 43(2): 49-56.	Population not relevant
116	Bauters, T., Robays, H., Van De Velde, V., Verlooy, J., Van Neck, A., Laureys, G., Benoit, Y. 2013. Restrospective analysis on the use of laxatives in children in palliative care Pediatric Blood and Cancer, 60(SUPPL. 3): 42.	Intervention/ comparison not relevant
119	Beardsmore, S., Fitzmaurice, N. 2002. Palliative care in paediatric oncology European Journal of Cancer, 38(14): 1900-1907.	Population not relevant
123	Bendle, Lizzie, Laddie, Joanna 2019. Symptomatic palliative care for children with neurodisability Paediatrics and Child Health (United Kingdom), 29(10): 431-435.	Study design not relevant
125	Bennett, M. B. 1973. Care of the dying South African medical journal = Suid-Afrikaanse tydskrif vir geneeskunde, 47(34): 1558-60.	Study design not relevant
128	Benze, G., Geyer, A., Alt-Epping, B., Nauck, F. 2012. [Treatment of nausea and vomiting with 5HT3 receptor antagonists, steroids, antihistamines, anticholinergics, somatostatinantagonists, benzodiazepines and cannabinoids in palliative care patients : a systematic review] Behandlung von ubelkeit und erbrechen mit 5HT3-antagonisten, steroiden, antihistaminika, anticholinergika, somatostatinanaloga, benzodiazepinen und cannabinoiden bei palliativpatienten : ein systematisches review., 26(5): 481-99.	Study design not relevant
152	Bonertz, Lori, Dyck, Nancy, Kantz, Valerie 2007. Provision of methadone infusion by a community pharmacy for a pediatric patient requiring palliative care Canadian Pharmacists Journal, 140(5): 298.	Population not relevant
177	Brown, Sarah, Davies, Natalie, Heather, Nicky, Cole, Caroline, Smyth, Enda, Batra, Akshay, Beattie, R. M. 2017. Long term outcome of intestinal rehabilitation in children over a period of 15 years-a single centre experience Journal of Pediatric Gastroenterology and Nutrition, 64(Supplement 1): 858-859.	Intervention/ comparison not relevant
190	Cairns, P. A. 2020. Decision-making in neonatal intestinal failure:palliative or active care? Archives of Disease in Childhood, 105(SUPPL 1): A117.	Population not relevant
192	Cameron, Jean-Christy F., Vaillancourt, Regis, Major-Cook, Nathalie, Boland, Margaret, Zucker, Marc, Lariviere, Doris 2013. Clinical recovery of chronic intestinal pseudo-obstruction with cisapride in a complex pediatric patient The American journal of hospice & palliative care, 30(4): 403-5.	Intervention/ comparison not relevant
195	Candy, Bridget, Jones, Louise, Vickerstaff, Victoria, Larkin, Philip J., Stone, Patrick 2018. Mu-opioid antagonists for opioid-induced bowel dysfunction in people with cancer and people receiving palliative care The Cochrane database of systematic reviews, 6(#issue#): CD006332.	Study design not relevant
236	Cheung, H. M., Lam, H. S., Tam, Y. H., Lee, K. H., Ng, P. C. 2009. Rescue treatment of infants with intestinal failure and parenteral nutrition-associated	Population not relevant

	cholestasis (PNAC) using a parenteral fish-oil-based lipid Clinical Nutrition, 28(2): 209-212.	
254	Coad, Jane, Toft, Alex, Lapwood, Susie, Manning, Joseph, Hunter, Mark, Jenkins, Huw, Sadlier, Clare, Hammonds, Julie, Kennedy, Ailsa, Murch, Simon, Widdas, David 2017. Blended foods for tube-fed children: a safe and realistic option? A rapid review of the evidence Archives of disease in childhood, 102(3): 274-278.	Study design not relevant
256	Cockington, R. A., Vining, R. A. 1985. Prescribing for children. Part II. Symptomatic treatment Current Therapeutics, 26(11): 57-80.	Study design not relevant
305	Di Nardo, Giovanni 2019. New therapeutic options in children with chronic intestinal pseudo-obstruction Italian Journal of Pediatrics, 45(Supplement 3): #Pages#.	Study design not relevant
333	Duval, M., Wood, C. 2002. [Treatment of non-painful symptoms in terminally ill children] Traitement des symptomes non douloureux chez l'enfant en fin de vie., 9(11): 1173-8.	Study design not relevant
380	Feudtner, Chris, Freedman, Jason, Kang, Tammy, Womer, James W., Dai, Dingwei, Faerber, Jennifer 2014. Comparative effectiveness of senna to prevent problematic constipation in pediatric oncology patients receiving opioids: A multicenter study of clinically detailed administrative data Journal of Pain and Symptom Management, 48(2): 272-280.	Population not relevant
386	Flerlage, Jamie E., Baker, Justin N. 2015. Methylnaltrexone for Opioid-Induced Constipation in Children and Adolescents and Young Adults with Progressive Incurable Cancer at the End of Life Journal of palliative medicine, 18(7): 631-3.	Population not relevant
402	Friedrichsdorf, Stefan J., Foster-Barber, Audrey, Hauer, Julie, Tremonti, Nadia, Ullrich, Christina K. 2010. Advanced management of distressing non-pain symptoms in pediatric palliative care Journal of Pain and Symptom Management, 39(2): 328-329.	Intervention/ comparison not relevant
461	Goulet, Olivier, Ruemmele, Frank 2006. Causes and management of intestinal failure in children Gastroenterology, 130(2 Suppl 1): S16-28.	Population not relevant
468	Greenfield, Katie, Holley, Simone, Schoth, Daniel E., Harrop, Emily, Howard, Richard F., Bayliss, Julie, Brook, Lynda, Jassal, Satbir S., Johnson, Margaret, Wong, Ian, Liossi, Christina 2020. A mixed-methods systematic review and meta-analysis of barriers and facilitators to paediatric symptom management at end of life Palliative Medicine, 34(6): 689-707.	Study design not relevant
496	Hauer, Julie 2017. Feeding Intolerance in Children with Severe Impairment of the Central Nervous System: Strategies for Treatment and Prevention Children (Basel, Switzerland), 5(1): #Pages#.	Study design not relevant
514	Herrick, A. L. 1996. Advances in palliative care for the patient with scleroderma Current opinion in rheumatology, 8(6): 555-60.	Study design not relevant
532	Hotta, R., Natarajan, D., Thapar, N., Hotta, Ryo, Natarajan, Dipa, Thapar, Nikhil 2009. Potential of cell therapy to treat pediatric motility disorders Seminars in Pediatric Surgery, 18(4): 263-273.	Intervention/ comparison not relevant
583	Johnson, Liza-Marie, Spraker, Holly L., Coleman, Jamie L., Baker, Justin N. 2012. An unusual case of Ogilvie syndrome in a pediatric oncology patient receiving palliative care after failed treatment with neostigmine Journal of palliative medicine, 15(9): 1042-6.	Population not relevant
586	Jordan-Ely, Julie, Dobson, Kyla M., Appaduray, Shaun, Hynson, Jenny, Kornberg, Andrew J., Hutson, John M., Southwell, Bridget R. 2015. Management of severe faecal impaction in an adolescent with Duchenne muscular dystrophy (DMD) receiving palliative care Journal of paediatrics and child health, 51(3): 351-2.	Population not relevant
716	Lee, Noel, Wald, Arnold 2011. The pharmacokinetics, pharmacodynamics, clinical efficacy, safety and tolerability of linaclotide Expert opinion on drug metabolism & toxicology, 7(5): 651-9.	Study design not relevant

856	Moss, Jonathan, Dickerson, David, Nunnally, Mark, Jacobsohn, Eric 2011. Methylnaltrexone treats opioid-induced bowel dysfunction in three critically ill patients American Journal of Gastroenterology, 106(SUPPL. 2): S369.	Study design not relevant
915	Noritz, G., Jersak, T., Tumin, D., Fosselman, D., Humphrey, G. L., Testa, M. 2019. Defining end stage gastrointestinal failure in patients with neurologic impairment Developmental Medicine and Child Neurology, 61(Supplement 3): 130.	Intervention/ comparison not relevant
917	Novak, Chris, Hogg, Amanda, Sue, Kyle, Davies, Dawn 2021. Peripherally acting mu-opioid receptor antagonists for treatment of opioid-induced constipation in children Paediatrics & child health, 26(2): e105-e109.	Population not relevant
966	Pawliuk, Colleen, Widger, Kim, Dewan, Tammie, Brander, Gina, Brown, Helen L., Hermansen, Anne-Mette, Gregoire, Marie-Claude, Steele, Rose, Siden, Harold Hal 2020. Scoping review of symptoms in children with rare, progressive, life-threatening disorders BMJ supportive & palliative care, 10(1): 91-104.	Study design not relevant
967	Peck, S. N., Altschuler, S. M. 1992. Pseudo-obstruction in children Gastroenterology nursing : the official journal of the Society of Gastroenterology Nurses and Associates, 14(4): 184-8.	Study design not relevant
980	Pettit, Kevin A., Beardmore, Daniel 2020. Constipation and asymmetric rectal tone in a 10-month-old girl Pediatrics in Review, 41(#issue#): S79-S81.	Population not relevant
996	Portnoi, M. V., Tsarev, N. I. 1969. [Elongated sigmoid colon] Udlinennaia sigmovidnaia kishka., 102(1): 46-50.	Population not relevant
1053	Roy, A., Simon, G. B. 1987. Intestinal obstruction as a cause of death in the mentally handicapped Journal of mental deficiency research, 31 (Pt 2)(#issue#): 193-7.	Intervention/ comparison not relevant
1072	Samuels, L. A. 2009. Pharmacotherapy update: hyoscine butylbromide in the treatment of abdominal spasms Clinical Medicine: Therapeutics, #volume#(1): 647-655.	Study design not relevant
1077	Santucci, Gina, Mack, Jennifer W. 2007. Common gastrointestinal symptoms in pediatric palliative care: nausea, vomiting, constipation, anorexia, cachexia Pediatric clinics of North America, 54(5): 673-x.	Study design not relevant
1093	Schuffler, M. D., Rohrmann, C. A., Chaffee, R. G., Brand, D. L., Delaney, J. H., Young, J. H. 1981. Chronic intestinal pseudo-obstruction. A report of 27 cases and review of the literature Medicine, 60(3): 173-96.	Study design not relevant
1107	Sewell, Mathew D., Eastwood, Deborah M., Wimalasundera, Neil 2014. Managing common symptoms of cerebral palsy in children BMJ (Online), 349(#issue#): g5474.	Intervention/ comparison not relevant
1116	Shaw, Tressia M. 2012. Pediatric palliative pain and symptom management Pediatric Annals, 41(8): 329-334.	Intervention/ comparison not relevant
1200	Tabbers, Merit M., Boluyt, Nicole, Berger, Marjolein Y., Benninga, Marc A. 2011. Nonpharmacologic Treatments for Childhood Constipation: Systematic Review Pediatrics, 128(4): 753-761.	Study design not relevant
1231	Thapar, N., Burns, A. 2012. Use of enteric nervous stem cells to treat motility disorders: Ready for prime time? Neurogastroenterology and Motility, 24(SUPPL.2): 15.	Study design not relevant
1241	Torres, Clarivet, Dussan, Monica, Sandler, Anthony, Zavosky, Patricia, Parvathi, Mohan 2009. A new intestinal care program (ICP) at children's national medical center: Another year of experience Pediatric Transplantation, 13(SUPPL. 1): 47.	Study design not relevant
1263	Uzcategui Arauz A, Arias Guzman Y. Jaen D. 1995. Chronic constipation. Use of cisapride Estrenimiento cronico. Uso del cisapride, 49(3): 218.	Population not relevant
1322	Westfal, Maggie L., Goldstein, Allan M. 2017. Pediatric enteric neuropathies: diagnosis and current management Current Opinion in Pediatrics, 29(3): 347-353.	Study design not relevant

1331	Wiseman, L. R., Faulds, D. 1994. Cisapride. An updated review of its pharmacology and therapeutic efficacy as a prokinetic agent in gastrointestinal motility disorders Drugs, 47(1): 116-52.	Study design not relevant
1332	Wiskin, A. E., Cullen, M., Pidgeon, C., Beattie, R. M., Cole, C., Owens, D. R., Burge, D. M. 2011. Home parenteral nutrition in children: 10 year experience from a single centre Proceedings of the Nutrition Society, 70(OCE5): E289.	Population not relevant
